# Supplementary material for: Human antimicrobial peptide, LL-37, induces non-inheritable reduced susceptibility to vancomycin in Staphylococcus aureus
Source: Sci Rep. 2020 Aug 4;10:13121. doi: 10.1038/s41598-020-69962-4 (PMC7403302; doi:10.1038/s41598-020-69962-4)
Supplement: Supplementary file 2 — Supplementary Figures. [file 41598_2020_69962_MOESM2_ESM.docx]

## Supplementary figures

## Human antimicrobial peptide, LL-37, induces non-inheritable reduced susceptibility to vancomycin in *Staphylococcus aureus*

Cathrine Friberg, Jakob Haaber, Martin Vestergaard, Anaëlle Fait, Veronique Perrot, Bruce Levin & Hanne Ingmer

**Figure S1. LL-37 effect on maximum growth rate.**

From the growth experiments as described in Materials and Methods, the maximum growth rates were determined for *S. aureus*cultures pre-exposed to LL-37 (0 to 20 µg/ml) for 1 h before being exposed to 0 or 0.75 µg/ml vancomycin. Error bars represent s.d. of 3 biological replicates each containing 3 technical replicates.


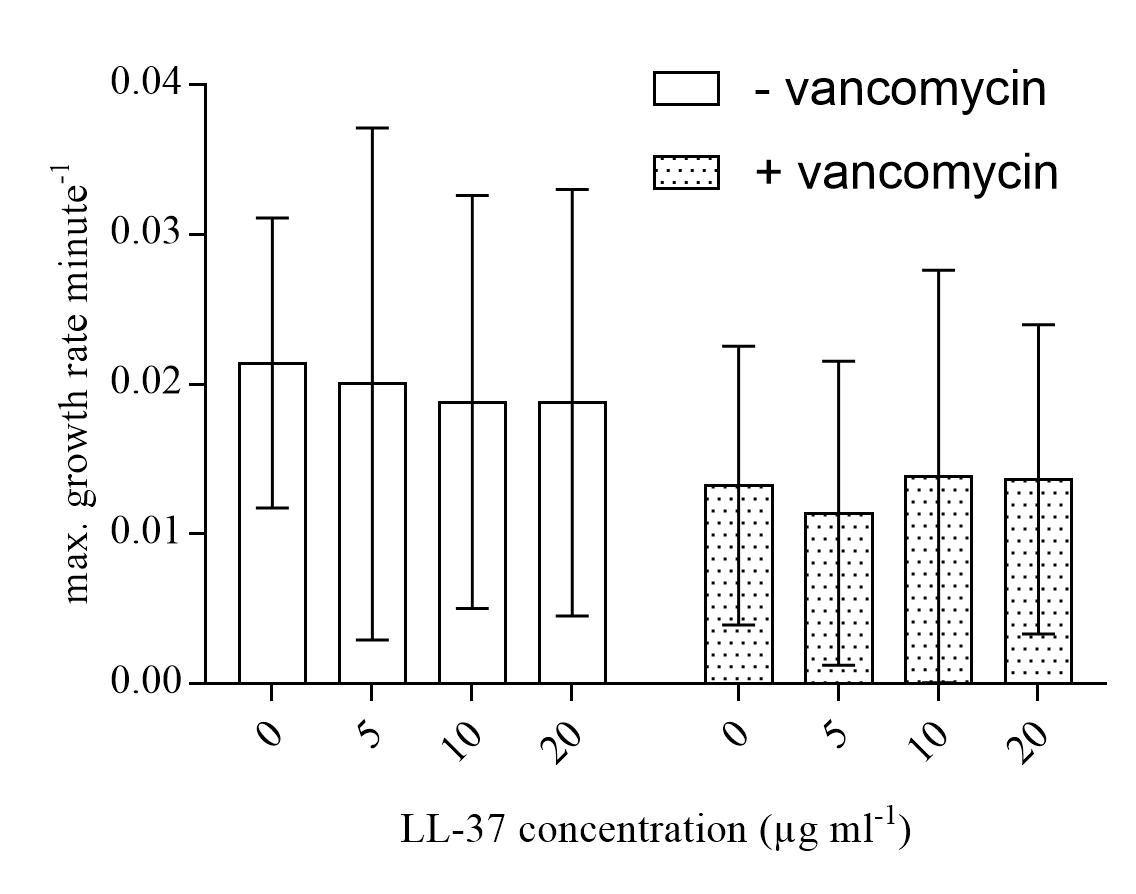


**Figure S2.** A) Hill function pharmacodynamics and B) the relationship between the concentration of the antibiotic and the rate of kill of bacteria with an antibiotic. The parameters of these functions and kill dynamics are the same as those in used in Figure 5 to illustrate the treatment dynamics anticipated with different concentrations of antibiotics and the MICs of these drugs.
